# Empirically derived dietary patterns through latent profile analysis among Brazilian children and adolescents from Southern Brazil, 2013-2015

**DOI:** 10.1371/journal.pone.0210425

**Published:** 2019-01-08

**Authors:** Adriana Soares Lobo, Maria Alice Altenburg de Assis, Danielle Biazzi Leal, Adriano Ferreti Borgatto, Francilene Kunradi Vieira, Patricia Faria Di Pietro, Emil Kupek

**Affiliations:** 1 Postgraduate Program in Nutrition, Center for Health Sciences, Federal University of Santa Catarina, Florianópolis, SC, Brazil; 2 Postgraduate Program in Physical Education, Sports Center, Federal University of Santa Catarina, Florianópolis, SC, Brazil; 3 Department of Informatics and Statistics, Technological Center, Federal University of Santa Catarina, Florianópolis, SC, Brazil; 4 Department of Public Health, Center for Health Sciences, Federal University of Santa Catarina, Florianópolis, SC, Brazil; Leibniz Institute for Prevention Research and Epidemiology BIPS, GERMANY

## Abstract

The aims of this study were to identify dietary patterns (DPs) of children and adolescents participating in three cross-sectional surveys (2013–2015) and to test their associations with sociodemographic variables, physical activity (PAS), screen-based sedentary activity (SA), and weight status. One-day data were obtained from 5,364 schoolchildren (7–12 years) from public schools of Florianopolis (South of Brazil), using the validated questionnaire Web-CAAFE (Food Intake and Physical Activity of Schoolchildren). DPs were derived from the frequency of daily consumption of 32 foods/beverages by latent profile analysis. Multinomial logistic regression analysis was used to estimate the association of the DPs with sociodemographic variables, physical activity, screen activity and weight status. ‘Traditional’, ‘Monotonous’, and ‘Mixed’ DPs were identified. The percentages of children and adolescents within these profiles were 41.3, 36.3, and 22.4%, respectively. Children and adolescents in the highest tertiles of both PAS and daily frequency of SA had a higher probability to present a ‘Mixed’ DP compared to peers with less PAS and SA. Children and adolescents who reported having a school meal were significantly more likely to present the ‘Traditional’DP, while boys who did not report having a school meal had a higher probability to present the ‘Monotonous’ DP. The DPs were not associated with the year of survey, age, family income, or weight status.

## Introduction

Surveillance and monitoring of health-related behaviors are critical to inform and develop preventive health policies, actions, laws and regulations [1,2]. Monitoring dietary intake and others health behaviors in childhood and adolescence is particularly important since these are crucial stages in the formation of eating habits and their maintenance in adulthood [3]. The need to monitor eating habits among young people has intensified in recent years due to the growing epidemic of overweight worldwide [4], including Brazil [5].

In Brazil, there are national surveillance systems for adolescents [6] and adults [7], but none for children under 10 years of age. To fill this gap, we developed an online nutritional surveillance system designed to collect periodic data on weight status (based on body mass index—BMI), food consumption, physical activities and sedentary behaviours, consumption of and satisfaction with school meals and physical activity classes. The data allow tracking of anthropometric measurements, dietary patterns and other health behaviours and their association with the weight status [8].

Recognizing that foods are eaten in combination creating complex interaction and correlation between nutrients and other food components, analysis of the whole dietary patterns (DPs), rather than individual nutrients or dietary component, has become increasingly important in examining diet and health outcomes [9–10]. DPs are identified by statistical dimensionality reduction procedures such as factor analysis, cluster analysis, reduced rank regression, latent profile analysis (LPA) and latent class analysis (LCA), which also provide means of identifying at-risk population subgroups [9–12], including in the early school age (6–11 years) [12–14].

Conceptually, LCA and LPA are similar to cluster analysis because they classify individuals into classes. However, because LCA and LPA are model based methods, it is possible to use goodness-of-fit tests to help determine the appropriate number of classes (i.e., dietary patterns) [15] for a variety of risk behaviours including eating behaviour [11,14,16]. LPA and LCA models estimate the probabilities of identified classes and probabilities of responses for each indicator, conditional on class membership [16].

Although DPs have been identified in cross-sectional studies in Brazilian children and adolescents [14,17–20], few studies assessed how the patterns change with time [11,21]. None of them used data from a nutritional surveillance system. Furthermore, information concerning DPs and related health behaviors among Brazilian children, such as physical activity and sedentary behavior, is scarce [14,22].

The aims of this study were: 1) to identify DPs by LPA in children and adolescents attending public schools in Florianopolis, Brazil, over three survey years (2013, 2014 and 2015), and 2) to test the associations of DPs with sociodemographic variables, physical activity, screen activity and weight status.

## Methods

### Study population and sampling design

This study was conducted as a part of nutritional surveillance of 7-12-y-old schoolchildren attending 2nd to 5th grades of elementary public schools in Florianopolis, (Brazil) from August to October in 2013, 2014 and 2015. Florianopolis is the capital of the state of Santa Catarina, in South of Brazil. Its population comprises 421,240 inhabitants, of which 11% are aged between 6 and 14 years. In the 2010 national census, Florianopolis presented a Human Development Index of 0.847 (the highest among Brazilian state capitals), a Gini Index for Income Inequality of 0.54, an infant mortality rate of 10.8/1000 live births, and the life expectancy at birth was 77.4 years [23].

The choice of the age range was based on the fact obesity diagnosis after the age of 6 years has a better predictive value of the adiposity status in later childhood and adulthood [24]. In addition, this age range is amenable to acquiring healthy eating habits in conjunction with other adaptations to the school environment, thus making this age of particular interest for surveillance and prevention programs.

### Sample size

The target population was all schoolchildren from 2nd to 5th grades in the municipal elementary schools provided with a computer room and Internet access (34 out of 37 schools in 2013, 34 out of 36 schools in 2014, and 35 out of 36 schools in 2015). Primary sampling units were eligible classrooms (2nd to 5th grades) which were randomly selected from the complete list of schools with computer rooms provided by the municipal educational authority. In 2013, 6,946 students were enrolled in 34 public schools; in 2014 there were 7,120 students in 34 schools, and in 2015 there were 7,174 students in 35 schools [25].

Sample size estimation was performed for each survey year separately based on the following parameters: expected overweight (including obesity) prevalence of 34% [26,27], a margin of error of ±3 percentage points and confidence level of 95%. Considering that the sample was clustered by class, in each school, a design effect of 1.5 was used. As a result, minimum sample sizes for the survey years 2013, 2014 and 2015 were estimated at 1,263, 1,266 and 1,268 students, respectively. A 30% safety margin was added for expected non-response due to not providing signed consent to participate in the survey and these numbers were increased to 1,804, 1,809 and 1,811, in the same order.

Between-school variation is an important source of the variation for several well established factors associated with food intake, such as family income and education which tend to be more homogenous within a school catchment area. Therefore, the sampling design opted to maximize this source at the cost of reducing between-class variation within schools, known to be of lesser magnitude from previous studies of the same population [26,27]. This rationale was operationalized by randomly selecting only one class for each grade of interest from the complete list of eligible classes within participating schools.

In every sampled class, all children with no mental or visual disabilities were invited to participate. In total, 9,100 children were invited (2,830 in 2013, 2,928 in 2014 and 3,342 in 2015). Both child and parental consent was obtained for 7,425 (81.6%) children. For the present study a total of 674 children with implausible dietary data (i.e., children who reported less than four food items per day or out of the mean ± 3 standard deviation (SD) interval, assuming a Poisson distribution for food frequency reports) and 372 children with missing data for body weight and height were excluded from analysis. In addition, a total 1,015 children who were tracked over the three survey years were also excluded, aiming to keep the cross-sectional analysis of the study. So the final sample included 5,364 schoolchildren (1,942 in 2013, 1,520 in 2014 and 1,902 in 2015), both sexes, aged 7–12 years.

This study was conducted according to the guidelines set out in the Code of Ethics of the World Medical Association (Declaration of Helsinki) and all procedures involving human subjects were approved by the Human Studies Committee of the Federal University of Santa Catarina.

### Measurements of dietary intake, physical activity and screen-based sedentary activities

Data were obtained using the Web-CAAFE (Food Intake and Physical Activity of Schoolchildren), a web-based self-reported questionnaire designed for use in the school settings in order to help public health and education professionals to evaluate schoolchildren from the 2nd to the 5th grade regarding: (i) weight status based on BMI, food consumption, physical activity and sedentary behaviors; (ii) assessment of school compliance and children’s acceptability of the National School Meals Program; (iii) participation in and satisfaction with physical education classes at school [8]. The Web-CAAFE examines food consumption, physical activity and sedentary behavior during the previous day (24-h recall). Usability tests showed very good acceptability and child capacity to understand and respond to Web-CAAFE [8]. Validity tests of the food consumption section, using direct observation at school meals as the reference method, showed 43% matches, 29% intrusions and 28% omissions [28], placing this questionnaire’s accuracy close to that of other similar instruments [29–30]. Self-reported sedentary behaviors and physical activity were also validated in a sample of schoolchildren that were directly observed during the school break time [31]. Web-CAAFE was also proved to be a valid questionnaire for screening compliance with dietary recommendations in medium-size and sometimes even smaller groups of children (e.g. a classroom) [32].

The questionnaire consists of three sections: registration, food consumption, and physical activity and sedentary behaviors. The registration section collects information about respondents’ name, sex, weight, height, age, date of birth and the school attendance schedule (morning or afternoon). The children’s weight and height measurements were taken by trained researchers and recorded in the school diary of each child to facilitate their registration [8]. The food consumption section of the Web-CAAFE comprises six daily eating occasions ordered chronologically (breakfast, mid-morning snack, lunch, afternoon snack, dinner, and evening snack). For each event, 32 images (icons) of foods/beverages or food groups are presented on the computer screen, including healthy and unhealthy items: rice, vegetables (such as carrots, pumpkin and broccoli), green leaves, vegetable soup, beans (cooked), manioc flour, maize/potatoes, pasta, instant pasta, French fries, beef/poultry, sausages, eggs (fried, boiled or omelet), fish/seafood, fruits (all kinds of traditional Brazilian fruits such as bananas and oranges), bread/biscuits, cheese bread, cream cookies, breakfast cereal, porridge, cheese, milk and coffee, milk, yoghurt, chocolate milk, fruit juices, sodas, sweets (such candies, chocolate bars, ice cream, cakes with icing), chips, pizza/hamburger/hot-dog, nuggets and cakes [28].

Like other instruments developed for children [33–35], the Web-CAAFE was not designed to provide an estimate of total energy intake or global food intake but to investigate the markers of (un)healthy foods related to weight status. The food items were chosen in order to take into account the food patterns of children of this age group, the food presented in school menus and the food recommended in the guidelines for Brazilian population [36]. In addition, four questions regarding school meals were asked, including “Did you have a school meal yesterday?” [8].

The physical activity section was divided into the three parts of the day (morning, afternoon, and evening); for each of them, 32 leisure activities, sports, home chores and sedentary activities were presented (basketball/volleyball, catch one, soccer, running, martial arts, tennis, dancing, table-tennis, marbles, hopscotch, jumping rope, gymnastics, swimming, cycling, rollerblading/skateboarding, surfing, kite flying, dodgeball, hide-and-sick, playing with the dog, studying/reading/drawing, board games, playing with dolls, playing with toy cars, watching TV, listening to music, using smartphone/tablet, using computer, play videogames, wash dishes, sweep) [8,31].

In the present study, physical activities were assigned a metabolic equivalent (METs) value using the Compendium of Energy Expenditures for Youth [37]. For each activity, a score was created multiplying the METs by the daily frequency (ranging from 0 to 3). The subject's physical activity scores (PAS) was the sum over all scores. PAS was categorized into tertiles (the first tertile was defined as lowest, second tertile as intermediary and third tertile as highest PAS). The daily frequency of screen-based sedentary activities (SA) reported during morning, afternoon and evening (watching television, playing video game, using a computer and tablet/cell phone) was also determined for each child and categorized into tertiles (the first tertile was defined as lowest, second tertile as intermediary and third tertile as highest SA). Tertiles of PAS and daily frequency of screen activities (SA) were combined into an exposure variable (combination of PAS and SA tertiles) with nine categories.

Web-CAAFE was applied in the school computer room once for every child and the day at which the questionnaire was assessed differed between children. This strategy was used in order to describe the daily variability of dietary intake and physical activity over school days (Monday to Thursday) and non-school days (Sunday and holydays) allowing for the analysis of these behaviors at the group level. As the Web-CAAFE was applied in the school setting and there was no school on Saturdays and Sundays it was not possible to obtain data representing food consumption, physical activity and sedentary behavior for Fridays and Saturdays.

### Anthropometric measurements

Measurements of the children’s weight and height were performed by ten trained physical education teachers, following standard techniques [38] and taken in lightly dressed barefoot children. Theoretical and practical workshops on measurement techniques were previously held in order to standardize anthropometric measurements. Weight was measured with a digital 180 kg scale (Marte, model PP, 50 g precision). Height was measured with a portable stadiometer (Alturexata, 1 mm precision). The children`s weight and height measurements were taken once, on the same day they answered the Web-CAAFE.

Body mass index (BMI) was computed as weight (kg) divided by height squared (m). Age- and sex-specific BMI z-scores were calculated according to the World Health Organization–WHO [39]. The weight status of children was categorized as either non-overweight (thinness and normal weight) or overweight including obesity.

### Family income

Family income was not available directly from the parents. Instead, average census sector income of the school location area was available from the Brazilian Institute of Geography and Statistics (IBGE) [40] and used as proxy for the family income because the family residential address determined the school a child was assigned to attend. The variable was categorised in tertiles (lowest, middle and highest family income).

### Statistical analysis

Sociodemographic characteristics of the study population were described using frequencies and percentages as well as means and standard deviations (SD) for continuous variables. The latent profiles indicators were the frequencies of consumption of 32 food/beverage items, estimated as number of times per day. The maximum frequency per day was 6 assuming that only one serving was consumed on each of the six eating events. LPA was used to assign the individuals to the most likely latent profiles based on their food consumption. It uses maximum likelihood algorithms to identify underlying subgroups in the data that are qualitatively distinct [15,16]. LPA is applied to ordinal or continuous variables to identify unobserved (latent) groups of individuals based on the principle of conditional independence (the variables are assumed uncorrelated within each class) [15].

Model fit was assessed using the Akaike information criterion (AIC), Bayesian information criterion (BIC) and Sample-size adjusted Bayesian information criterion (SS-ABIC), with lower values indicating a better fit. The Lo-Mendell-Rubin probability (LMR prob) was also calculated. It tested the parsimony of the current model against a model with one fewer class (e.g. 3 *vs*. 2 classes), providing the probability (*p-*value) that the current model is not an improvement on the model containing one class less [41,42]. LPA was performed separately for each year and with the pooled data.

Posterior probability and its precision were calculated for consuming a dietary item conditional on the latent class membership and divided by the sample average frequency of consumption (AFC). The latter can be considered an estimate of unconditional probability of consuming a dietary item, so that the ratio of the conditional and unconditional probability represents a deviation from the expected consumption frequency. This measure was denominated ratio to average frequency of consumption (RAFC) and was calculated for each pattern. RAFC and its 95% confidence intervals (CI) were calculated as normal deviates from the standard errors of the LPA posterior probability estimates. When the confidence interval includes the value of one, it is not statistically significant on the level selected (p<0.05).

Trends in DPs (latent profiles) over the survey years were assessed by using weighted linear regression with weights equal to the inverse of the RAFC point estimates’ variance. RAFC for each food item, within each latent profile, was the dependent variable and the survey year was the independent variable. Adjustments were performed by sex, children’s age (7–9 *vs*. 10–12 years), family income tertiles; combination of PAS and SA tertiles, self-reported consumption of a school meal (yes *vs*. no), the day of the week the food consumption was reported (school day *vs*. non-school day), and weight status categories (non-overweight *vs*. overweight including obesity).

Multinomial logistic regression analysis stratified by sex were used to estimate the children's probabilities to belong to each identified pattern. The exposure variables included in the multivariate models were: survey year (2013, 2014 or 2015), children’s age (7–9 *vs*. 10–12 years), family income tertiles; combination of PAS and SA tertiles, self-reported consumption of a school meal (yes *vs*. no), the day of the week the food consumption was reported (school day *vs*. non-school day), and weight status categories (non-overweight *vs*. overweight including obesity). Conditional posterior distribution ("marginal distributions") for each LPA was presented in terms of predicted probabilities with corresponding 95% CI, adjusted for all other covariates, and no statistical test of the null hypotheses was performed. The 95% CI was preferred as they convey the magnitude and variation of the effects analysed. Statistically significant differences between the independent variables’ levels within each latent dietary profile were verified by non-overlapping 95% CI.

Statistical software Mplus version 6.04 was used for LPA and Stata 12.0 (StataCorp, 2011) for descriptive statistics, Student’s *t*-test, Chi-square test and multinomial regression.

## Results

The final study population comprised 5,364 children and adolescents (2,636 girls and 2,728 boys) aged 7–12 years (mean ± SD = 9.1±1.3 years), with complete anthropometric, dietary, physical activity and screen-based sedentary activity data.

Table 1 summarizes characteristics of the participants by survey year. The proportion of boys was slightly higher (50.9%), except in 2014 (49.5%). Most of the children had 7–9 years old. The prevalence of overweight (including obesity) was 34.5% and it was higher in 2014 (37.3%). The majority of the children (78.1%) reported food consumption on a school day (versus non-school day) and 40.8% reported having a school meal, but both values were lower in 2014 survey (70.5% and 34.6%, respectively). The average monthly income of the families was R$ 2,029 (U$ 776) between 2013 and 2015.

**Table 1:** Descriptive characteristics of children and adolescents included in three annual surveys in Florianopolis, Brazil.

	2013 (N = 1,942)		2014 (N = 1,520)		2015(N = 1,902)		All (N = 5,364)	
Characteristics	N	%	N	%	N	%	N	%
**Sex**								
Boys	992	51.1	753	49.5	983	51.7	2,728	50.9
Girls	950	48.9	767	50.5	919	48.3	2,636	49.1
**Age (years)**								
7–9	1,145	59.0	847	55.7	1,214	63.8	3,206	59.8
10–12	797	41.0	673	44.3	688	36.2	2,158	40.2
**Weight status**^**a**^								
Non overweight	1,317	67.8	953	62.7	1,245	65.5	3,515	65.5
Overweight (including obesity)	625	32.2	567	37.3	657	34.5	1,849	34.5
**Day of the week recall**								
Not schoolday	342	17.6	449	29.5	383	20.1	1,174	21.9
Schoolday	1,600	82.4	1,071	70.5	1,519	79.9	4,190	78.1
**Having school meal**								
No	1,107	57.0	994	65.4	1,072	56.4	3,173	59.2
Yes	835	43.0	526	34.6	830	43.6	2,191	40.8
**PAS**^**b**^ **(**Mean±SD)	23.0±13.6		23.5±13.6		22.7±13.5		23.0±13.5	
**Screen activities**^**c**^ **(**Mean±SD)	2.3±2.3		2.3±2.1		2.4±2.0		2.3±2.1	
**Family income (R$)**^**d**^ **(**Mean±SD)	2,014±1,060		2,021±969		2,052±993		2,029±1,011	

N, total numbers of subjects; SD, standard deviation; R$, Reais (Brazilian currency).

^a^ WHO, 2007 [42]

^b^ PAS, physical activity score computed by multiplying the metabolic equivalent of each physical activity [40] by the daily frequency reported. The subject's PAS was the sum over all scores (PAS values range from 3.6 to 96.9)

^c^ Number of all self-reported screen-based sedentary activity self-reports (watching television, playing video game, using a computer or tablet/cell phone)

^d^ Monthly family income based on the school census sector; U$ = R$ 2,61 (average exchange rate between 2013 and 2015)

Goodness-of-fit measures for the number of LPA classes in each year and in the pooled data showed that the 3-class model was the best model and it was used in all subsequent analysis (S1 Table). Time trend analysis showed no statistically significant effect except for sausage consumption in the latent profile named “Mixed” (p = 0.037) (S2 Table). This finding enhanced the analysis of the pooled data as the major focus of the present study.

Table 2 presents the three latent profiles (or Dietary Patterns–DPs) expressed as RAFC and labeled according to the dominating food items in the total sample. The first DP was labeled “Traditional” and included 41.3% of the children. Compared to the whole sample average frequency of consumption (AFC), they had significantly higher frequency of consuming beans (cooked) (43%), rice (41%), manioc lour (18%), bread and biscuits (26%), beef/poultry (24%), fruits (26%), vegetables (42%), green leaves (49%), milk and coffee (36%) and milk (16%).

**Table 2:** Average frequency of consumption (AFC) and ratio to average frequency of consumption (RAFC) for each food item according to latent profile DPs of children and adolescents over the pooled 2013–2015 period in Florianopolis, Brazil (N = 5,364).

		Latent dietary patterns [N (% of children)]					
Food groups/food items	AFC^a^	Traditional [2,218 (41.3%)]		Monotonous [1,946 (36.3%)]		Mixed [1,200 (22.4%)]	
		RAFC^b^	*p-trend*^c^	RAFC^b^	*p-trend*^b^	RAFC^b^	*p-trend*^c^
**Beans (cooked)**	0.80	1.43 (1.39–1.48)*	0.729	0.44 (0.41–0.47)*	0.901	1.11 (1.06–1.17) *	0.588
**Cereals**							
Rice	1.02	1.41 (1.38–1.44)*	0.676	0.53 (0.50–0.55)*	0.980	1.00 (0.96–1.05)	0.470
Manioc flour	0.23	1.18 (1.08–1.28)*	0.291	0.26 (0.21–0.31)*	0.808	1.86 (1.70–2.02)*	0.347
Maize/potatoes	0.13	0.82 (0.71–0.94)*	0.555	0.45 (0.37–0.53)*	0.547	2.22 (1.97–2.46)*	0.823
Pasta	0.33	0.49 (0.43–0.54)*	0.166	1.23 (1.14–1.32)*	0.774	1.58 (1.45–1.70)*	0.696
Instant pasta	0.17	0.48 (0.41–0.56)*	0.682	1.21 (1.09–1.33)*	0.906	1.62 (1.44–1.80)*	0.222
Bread/biscuits	1.06	1.26 (1.22–1.29)*	0.748	0.76 (0.73–0.80)*	0.934	0.91 (0.86–0.96)*	0.172
Breakfast cereal	0.17	0.57 (0.49–0.65)*	0.225	0.74 (0.64–0.84)*	0.725	2.22 (2.01–2.43)*	0.883
Porridge	0.08	0.88 (0.73–1.02)	0.078	0.66 (0.53–0.80)*	0.894	1.78 (1.48–2.07)*	0.477
**Meats/eggs/fish and seafoods**							
Beef/poultry	0.80	1.24 (1.20–1.28)*	0.981	0.70 (0.66–0.73)*	0.901	1.05 (0.99–1.11)	0.178
Eggs	0.17	1.02 (0.91–1.13)	0.467	0.48 (0.40–0.56)*	0.077	1.81 (1.62–2.00)*	0.897
Fish/seafood	0.12	0.74 (0.63–0.84)*	0.307	0.71 (0.59–0.82)*	0.856	1.96 (1.73–2.20)*	0.625
**Fruits/vegetables**							
Fruits	0.46	1.26 (1.18–1.33)*	0.925	0.49 (0.45–0.54)*	0.357	1.35 (1.24–1.45) *	0.265
Vegetables	0.23	1.42 (1.31–1.53)*	0.642	0.15 (0.11–0.18)*	0.777	1.61 (1.46–1.76)*	0.402
Green leaves	0.23	1.49 (1.38–1.60)*	0.725	0.09 (0.06–0.11)*	0.590	1.58 (1.42–1.73)*	0.341
Vegetable soup	0.19	0.48 (0.41–0.55)*	0.617	1.10 (0.99–1.21)	0.994	1.79 (1.61–1.97)*	0.734
**Milk/milk products**							
Milk and coffee	0.44	1.36 (1.28–1.43)*	0.877	0.67 (0.62–0.71)*	0.507	0.93 (0.85–1.01)	0.198
Milk	0.23	1.16 (1.06–1.27)*	0.554	0.43 (0.36–0.49)*	0.317	1.63 (1.48–1.79)*	0.295
Yoghurt	0.39	0.83 (0.77–0.89)*	0.875	0.66 (0.60–0.72)*	0.433	1.86 (1.74–1.98)*	0.304
Cheese	0.13	1.13 (0.99–1.27)	0.585	0.32 (0.25–0.39)*	0.723	1.86 (1.62–2.10)*	0.600
**Salted snacks/fast-foods**							
Cheese bread	0.13	0.48 (0.39–0.56)*	0.799	0.48 (0.40–0.57)*	0.801	2.81 (2.54–3.07)*	0.451
French fries	0.23	0.27 (0.23–0.32)*	0.834	0.63 (0.55–0.70)*	0.391	2.95 (2.75–3.15)*	0.360
Chips	0.10	0.28 (0.21–0.34)*	0.836	0.68 (0.56–0.80)*	0.401	2.86 (2.57–3.14)*	0.161
Pizza/hamburger/hot-dog	0.24	0.17 (0.14–0.21)*	0.826	1.49 (1.38–1.60)*	0.923	1.74 (1.59–1.89)*	0.382
Sausages	0.26	0.94 (0.85–1.03)	0.629	0.64 (0.57–0.71)*	0.348	1.70 (1.55–1.86)*	0.037
Nuggets	0.06	0.32 (0.22–0.42)*	0.405	0.73 (0.55–0.90)*	0.384	2.71 (2.30–3.12)*	0.080
**Sweets**							
Cake	0.29	0.68 (0.62–0.75)*	0.765	0.83 (0.76–0.91)*	0.981	1.85 (1.71–1.99)*	0.191
Candies/chocolate/lollipops/ice cream	0.23	0.52 (0.45–0.58)*	0.191	0.91 (0.82–1.00)	0.990	2.04 (1.88–2.21)*	0.902
Cream cookies	0.46	0.75 (0.69–0.80)*	0.171	0.79 (0.73–0.85)*	0.557	1.80 (1.70–1.91)*	0.282
**Sugar-sweetened beverages**							
Fruit juices	0.55	1.06 (1.00–1.12)	1.000	0.60 (0.55–0.65)*	0.342	1.54 (1.44–1.64)*	0.644
Soda	0.51	0.42 (0.38–0.46)*	0.556	1.07 (1.00–1.14)	0.717	1.96 (1.85–2.07)*	0.297
Chocolate milk	0.47	0.84 (0.78–0.90)*	0.923	0.93 (0.87–1.00)	0.647	1.41 (1.31–1.50)*	0.723

AFC: Average Frequency of Consumption; RAFC: Ratio to Average Frequency of Consumption

^a^AFC: Sample average frequency of consumption

^b^RAFC: The ratio of the mean food intake among the children belonging to each latent pattern and the AFC

^c^ P-trend calculated by weighted regression on year-specific RAFC point estimates

*When 95% confidence interval does not include the value of one, it is statistically significant.

The second DP was labeled “Monotonous” and comprised 36.3% of the children, with significantly higher frequency of consuming pasta (23%), instant pasta (21%) and pizza/hamburger/hot-dog (49%) compared to the sample AFC. Another distinctive feature was the lower frequency of consuming several food items, including beans (cooked) (-56%), manioc flour (-74%), maize/potatoes (-55%), fruits (-51%), vegetables (-85%), milk (-57%), cheese (-68%).

The third DP included 22.4% of the children with significantly higher frequency of eating a variety of foods, such as maize/potatoes (122%), breakfast cereals (122%), cheese bread (181%), French fries (195%), chips (186%), nuggets (171%), candies/chocolate/lollypops/ice cream (104%), soda (96%). Other food items whose consumption exceeded at least 30% of the sample average were manioc flour, pasta, instant pasta, porridge, eggs, fish/seafood, fruits, vegetables, green leaves, vegetables soup, milk, cheese, yoghurt, pizza/hamburger/hot/dog, sausages, cream cookies, cake, fruit juices and chocolate milk. This DP was labeled "Mixed".

The “Traditional DP” was presented in a higher proportion of girls (55%) and in children who reported food consumption on schooldays (82.5%), as well as by those having a school meal (45.1%). More boys (56.7%) than girls and more children with lower PAS (34.6%) belonged to the “Monotonous DP”. The proportion of children in the highest tertiles of PAS and SA (45.3% and 34%, respectively), and those who reported food consumption on non-school days (25.5%) was higher in the “Mixed DP” (data not shown).

The relationship between the latent profiles and the independent variables was investigated by multinomial logistic regression and presented as fully adjusted marginal probabilities for each category (Table 3). Among boys, those in the lowest tertile of PAS and in the highest tertile of SA had 42.1% probability to present a Monotonous DP (95% CI 34.2–50.0), while boys in the highest tertile of PAS and in the lowest tertile of SA had only 29.2% (95% CI 23.3–35.1)–a statistically significant reduction by more than 30%. However, those in the highest tertiles of both PAS and SA had the probability of belonging to the Mixed DP 2.13 times higher compared to those in the lowest tertile of PAS and in the highest tertile of SA (36.8%, 95% CI 31.2–42.3 vs. 17.3%, 95% CI 11.1–23.5).

**Table 3:** Probability (%) of belonging to a latent profile at different levels of the independent variables based on multinomial logistic regression, stratified by sex, of the children and adolescents evaluated in 2013–2015, Florianopolis, Brazil.

	Boys (2,728; 50.9%)			Girls (2,636; 49.1%)		
	Traditional (999; 36.6%)	Monotonous (1,104; 40.5%)	Mixed (625; 22.9,%)	Traditional (1,219; 46.2%)	Monotonous (842; 31,9%)	Mixed (575; 21,8%)
Characteristics	Probability (95% CI)			Probability (95% CI)		
**Year of survey**						
2013	35.9 (32.9–38.8)	40.9 (37.9–43.9)	23.2 (20.7–25.8)	47.8 (44.6–50.9)	31.8 (28.9–34.7)	20.4 (17.9–22.8)
2014	35.8 (32.4–39.3)	39.4 (35.9–42.8)	24.8 (21.8–27.8)	44.2 (40.6–47.7)	33.5 (30.2–36.8)	22.4 (19.6–25.2)
2015	38.0 (34.9–41.0)	40.9 (37.9–43.9)	21.1 (18.7–23.6)	46.4 (43.2–49.6)	30.8 (27.9–33.8)	22.8 (20.2–25.4)
**Age (years)**						
7–9	34.7 (32.4–37.0)	42.2 (39.9–44.6)	23.1 (21.1–25.1)	44.3 (41.9–46.8)	32.7 (30.5–35.0)	22.9 (20.9–25.0)
>10	39.5 (36.6–42.4)	37.8 (34.9–40.6)	22.7 (20.3–25.0)	49.1 (46.1–52.1)	30.6 (27.9–33.4)	20.3 (18.0–22.6)
**Family income (tertiles)**^**a**^						
Lowest	36.9 (33.8–39.9)	39.7 (36.7–42.7)	23.4 (20.8–25.9)	47.0 (43.8–50.2)	31.8 (28.8–34.7)	21.2 (18.7–23.7)
Medium	34.0 (30.9–37.2)	40.7 (37.6–43.9)	25.2 (22.5–28.0)	42.9 (39.6–46.1)	33.5 (30.5–36.6)	23.6 (20.9–26.3)
Higher	39.1 (35.8–42.3)	41.1 (38.0–44.4)	19.9 (17.4–22.6)	48.9 (45.5–52.3)	30.4 (27.4–33.5)	20.6 (18.0–23.3)
**Combination of PAS *and* SA tertiles**^**b**^						
Lowest PAS x Lowest SA	36.9 (30.2–43.6)	52.5 (45.6–59.4)	10.6 (6.3–14.8)	46.6 (41.0–52.2)	41.8 (36.2–47.4)	11.6 (8.0–15.2)
Lowest PAS x Intermediary SA	39.4 (34.5–44.2)	49.8 (44.9–54.7)	10.8 (7.7–13.9)	51.1 (45,9–56.2)	40.2 (35.2–45.2)	8.7 (5.8–11.6)
Lowest PAS x Highest SA	40.6 (32.6–48.5)	42.1 (34.2–50.0)	17.3 (11.1–23.5)	47.8 (37.8–57.8)	33.6 (24.2–43.0)	18.6 (10.8–26.4)
Intermediary PAS x Lowest SA	32.2 (27.2–37.3)	47.1 (41.7–52.6)	20.7 (16.2–25.1)	48.4 (43.1–53.7)	31.5 (26.6–36.5)	20.1 (15.8–24.3)
Intermediary PAS x Intermediary SA	40.9 (34.7–47.1)	40.7 (34.5–46.9)	18.4 (13.5–23.3)	48.4 (42.6–54.3)	30.2 (24.8–35.6)	21.4 (16.5–26.2)
Intermediary PAS x Highest SA	39.2 (32.1–46.3)	36.7 (29.6–43.7)	24.2 (20.6–25.2)	41.4 (33.8–49.1)	32.1 (24.8–39.3)	26.5 (19.6–33.4)
Higher PAS x Lowest SA	35.0 (28.8–41.1)	29.2 (23.3–35.1)	35.8 (29.6–42.0)	45.7 (39.3–52.0)	19.9 (14,7–25.1)	34.4 (28.3–40.6)
Higher PAS x Intermediary SA	36.3 (30.9–41.7)	27.6 (22.6–32.6)	36.1 (30.7–41.4)	46.4 (40.2–52.5)	17.8 (13,0–22.6)	35.8 (29.9–41.8)
Higher PAS x Highest SA	34.1 (28.6–39.5)	29.2 (23.9–34.4)	36.8 (31.2–42.3)	37.3 (30.9–43.7)	27.0 (21.1–32.9)	35.7 (29.3–42.1)
**Day of the week recall**						
Non-school days	31.1 (27.0–35.2)	42.3 (38.1–46.5)	26.5 (22.7–30.3)	37.4 (33.1–41.7)	35.2 (31.1–39.4)	27.4 (23.4–31.3)
School days	38.1 (36.0–40.2)	39.9 (37.9–42.0)	22.0 (20.3–23.7)	48.7 (46.5–50.9)	31.0 (28.9–33.0)	20.3 (18.6–22.0)
**Having school meal**						
No	34.4 (32.0–36.8)	43.3 (40.9–45.7)	22.3 (20.3–24.3)	45.3 (42.6–47.9)	33.9 (31.4–36.3)	20.8 (18.7–22.9)
Yes	40.0 (37.0–43.1)	36.0 (33.0–39.0)	23.9 (21.3–26.6)	47.6 (44.5–50.6)	29.2 (26.4–32.0)	23.2 (20.6–25.8)
**Weight status**^**c**^						
Non-overweight	36.9 (34.6–39.1)	39.1 (36.9–41.3)	24.0 (22.2–25.9)	44.9 (42.6–47.2)	32.9 (30.8–35.1)	22.2 (20.3–24.0)
Overweight (including obesity)	36.2 (33.2–39.2)	42.9 (39.9–46.0)	20.9 (18.4–23.4)	48.9 (45.6–52.2)	30.0 (27.0–32.9)	21.1 (18.6–23.7)

CI: Confidence Interval

^a^Family monthly income based on the school census sector; ^a^ U$ = R$ 3,20 (October 2016 exchange rate)

^b^PAS = physical activity scores, SA = screen-based sedentary activities self-reports

^c^WHO, 2007[42]

Likewise, girls in the highest tertiles of both PAS and SA had more than three times the probability of belonging to the “Mixed DP” (35.7%, 95% CI 29.3–42.1 28.2–40.1 vs. 11.6%, 95% CI 8.0–15.2) and were 35.4% less likely to present the "Monotonous DP” (27.0%, 95% CI 21.1–32.9 vs. 41.8%, 95% CI 36.2–47.4) compared to their peers with less PAS and SA.

Boys and girls who reported food consumption on school days were significantly more likely to present the “Traditional DP” compared to children who reported consumption on non-school days (boys: 38.1%, 95% CI 36.0–40.2 vs. 31.1%, 95% CI 27.0–35.2; girls: 48.7%, 95% CI 46.5–50.9 vs. 37.4%, 95% CI 33.1–41.7). Girls who reported consumption on non-school days were significantly more likely to present the “Mixed pattern” (27.4%, 95% CI 23.4–31.3 vs. 20.3, 95% CI 18.6–22.0). Finally, boys who reported having a school meal were significantly more likely to present the “Traditional DP” compared to boys who did not report such meal (40.0%, 95% CI 37.0–43.1). On the other hand, boys who did not report having a school meal had a higher probability to present the “Monotonous pattern” (43.3%, 95% CI 40.9–45.7 vs 36.0%, 95% CI 33.0–39.0 35.2–40.8).

The year of the survey, children’s age, family income and weight status did not show statistically significant association with the DPs.

## Discussion

In the present study conducted with schoolchildren from public schools of a southern Brazilian city, we identified three DPs (“Traditional”, “Monotonous” and “Mixed”) which did not show increasing or decreasing trends over the survey years (2013–2015), except for one food item (sausage) in the Mixed DP. A higher proportion of boys presented the “Monotonous DP”, whereas a higher proportion of girls presented the “Traditional” pattern. The “Mixed” pattern was associated with higher PAS and SA in boys and girls. We also observed that the “Traditional DP” was associated with food consumption on school days (as opposed to non-school days) in both boys and girls, and with having a school meal in boys. Overweight (including obesity) did not show a statistically significant association with the patterns.

This is the first Brazilian study that used data from a surveillance system for monitoring weight status, dietary intake and physical activity of schoolchildren under 12 years of age, as provided by a validated web-based questionnaire. As periodic surveys are the principal source of time trends in weight status and associated lifestyle factors such as diet and physical activity, the present study provides a pilot for the surveillance system performance in other Brazilian regions whose schools are provided with computer equipment and Internet access.

Previous studies examining DPs in children and adolescents have used factor analysis, principal component analysis, cluster analysis or reduced rank regression [12,13,17–21,43,44]. We used LPA to identify unobserved homogenous groups based on dietary intake for a wide range of foods and beverages. To the best of our knowledge, this is the first study that used LPA to derive DPs in children, although LCA has already been used for binary indicators of time-of-day food consumption (yes *vs*. no) to derive DPs associated with obesity in 7-11-yrs old Brazilian children [14]. Both latent analysis are akin to cluster analysis, only LCA and LPA had their results compared to the average consumption of each food/food group to provide an easier interpretation of the DPs. In addition, the latent variables may account for unknown confounding variables such as food availability and preference. Such variables are often unavailable with food questionnaires and play a role similar to the random effects in mixed model regression analysis.

It is difficult to make direct comparisons between the present results and those of other studies due to the different ages examined, different methods used to derive DPs, and also because the patterns may be specific to the populations studied [10,45]. Gama, Carvalho and Chaves [17] used cluster analysis in a study conducted in 5-9-y-old children treated at a primary health care unit in the city of Rio de Janeiro (n = 356) and they identified six DPs: “Traditional Brazilian cooking”, “Modern food”, “Fried food”, “Sweets and soft drinks” and other poorly defined groups. Using data from 60,954 pupils attending the last year of elementary education in public and private schools and who completed the Brazilian National School-Based Adolescent Health Survey (PeNSE), Tavares et al. [43] identified three dietary patterns (“healthy”, “unhealthy” and “mixed patterns”), also by cluster analysis.

Some of the studies of DPs in children [17–19], adolescents [46] and adults [44] in Brazil have found a DP similar to the traditional pattern described in the present study. It presented healthy aspects of traditional Brazilian foods, which often included rice, beans, vegetables and bread. However, the current study did not find that normal weight children had greater adherence to the “Traditional” dietary pattern, whereas other studies had observed a protective effect of the “traditional” DP against overweight [46,47]. However, these reports used a different statistical approach (factor analysis) than the present study (latent class profiles), making comparisons difficult. The higher proportion of girls with a “Traditional DP” is in line with other studies in which this and other healthy DPs were more prevalent among females [47,48].

The “Traditional DP” was associated with food consumption reported on school days, i.e., on weekdays (as opposed to non-school days, on weekends and holydays) in both boys and girls. Previous research in children indicates that dietary quality is poorer on the weekends compared with weekdays, with significantly higher intakes of total sugars [49], sugar sweetened beverages, confectionery and lower consumption of fruit and vegetables [50]. DP analysis showed that Danish children consistently consumed less healthy foods on the weekend regardless of whether they followed a healthy or a processed food pattern during the week [50]. Changes in daily patterns such as not attending school on the weekend contribute significantly to changes in DPs of food consumption, patterns of physical activity and ultimately energy balance [51]. In addition, the Traditional DP was associated to having a school meal in boys. In Brazil, school meals are planned by nutritionists in accordance with the National School Feeding Program (*Programa Nacional de Alimentação Escolar*—PNAE), whose aim is to promote healthy eating habits in school [52]. The program was implemented in Brazil since 1955 for all children in public primary schools and has provided a variety of healthy diet recommendations, such as reducing the consumption of high fat processed foods, sodium and sugar in the school setting.

A “Monotonous” DP was described using factor analysis in 8–9 years old children in Viçosa, southeastern Brazil, which was characterized by high consumption of milk and chocolate powder [19]. In the present study, the “Monotonous DP” was characterized by higher frequency of consuming pasta, instant pasta and pizza/hamburger/hot-dog. It should be mentioned that the adherence to the “Monotonous DP” was higher among boys that did not report having school meals compared to those who did. The latest version of Brazilian Dietary Guidelines [53] advocate the consumption of a variety of fresh or minimally processed foods as the basis of a nutritionally balanced, tasty and culturally appropriate diet. Therefore, school meals seem to play an important role in promoting a healthy diet by creating opportunities to expand the food diversity and establish a reference for healthy eating.

Many studies have identified unhealthy patterns under a variety of labels (“unhealthy”, “processed foods”, “obesogenic” or “junk food” patterns) which include items such as fast foods, soft drinks, sweets, cakes, French fries, processed foods and other unhealthy foods [18,44,45]. The present study also identified a “Mixed” pattern characterized by the presence of both healthy and unhealthy foods, in line with other studies using different methodologies to derive DPs [44,54]. In addition, the children with this pattern also reported higher levels of both physical activity and screen activities. A review of 18 studies using data-driven methodologies to examine the clustering of diet, physical activity and sedentary behaviors in children and adolescents showed that healthy and unhealthy patterns clustered in a variety of ways that were both beneficial and deleterious to good health. These patterns differed across sociodemographic groups but were not consistently associated with overweight/obesity [55]. By the same token, the absence of a statistically significant association between weight status and dietary patterns in the present study may be due to complex interactions of dietary intake, physical activity, and sedentary behaviors. This finding underlines the difficulty to disentangle the health impact of these components in isolation from each other.

Further limitations of the present study stem from measurement errors, both random and systematic [56], present in all self-reported dietary assessment methods. An important source of the random error is within-person day-to-day variation in food intake [57]. Although the food questionnaire was applied on various days of the week, this study design does not allow estimating the within-person or intra-person variation, thus underestimating this variance component [58]. In addition, despite the fact that the dietary patterns based on one-day dietary recall as in the present study do not necessarily capture the usual dietary patterns at the individual level, this method is widely accepted to assess food intake at the population level [56, 59, 60]. The failure to account for day-to-day within-person variation in food intake decreased statistical power to detect true associations between dietary patterns and overweight/obesity [58]. Day-to-day variability in food consumption can be modeled if at least two days of recalls/records on nonconsecutive days are collected on a large sample of subjects [56, 60] as practiced in a national Brazilian survey [61], and even better with three-day food records as in the Avon Longitudinal Study of Parents and Children [12] or with seven-day records as in a Danish food survey [50].

Another limitation is that the amount of food consumed (e.g. serving size) and the time spent in physical and sedentary activities were not measured. Short-form FFQs for children avoid the difficulties associated with the assessments of portion size and simplifies the memory task by prompting only the most relevant diet markers of the previous day [62,63]. In addition, the closed list of activities simplifies the task of recall by prompting only the relevant types of PA performed on the previous day, and keeps the questionnaire relatively brief and easy to complete. Also, food consumption questionnaires based on one 24 h recall [14,21,64,65] or one day record [44,66] have all been used to determine empirical dietary patterns for children and adolescents. We also acknowledged that only one measurement of body weight and height was taken per child or adolescent, although trained researchers performed the measures.

As the study population did not include the children from private schools who have a better socioeconomic status, the present study results can only be extrapolated to the children from public schools. The generalizability of the findings to other Brazilian regions is also unknown and requires further investigation. Finally, the data are cross-sectional observations, which provide solid basis for estimating associations but generally do not allow inferring a causal relationship.

Notwithstanding these limitations, the study has several important strengths. It included 7–12 years-old children from over 92% of the municipal public schools in Florianopolis, Brazil, who answered an online questionnaire validated for this age group. To the best of authors’ knowledge, it is the largest study of this population age regarding dietary patterns and physical activity using data from repeated cross-sectional surveys. Additionally, a novel feature of the study was the use of LPA to derive DPs in children with standardized criteria to determine the number of classes. Finally, we highlight the potential of the present findings to guide school-based nutritional health interventions.

## Conclusion

In summary, LPA categorized schoolchildren in three non-overlapping DP groups denominated “Traditional”, “Monotonous” and “Mixed”. Schoolchildren in the highest tertiles of physical activity and sedentary behavior scores were significantly more likely to present a Mixed DP. The “Traditional” DP was associated with the report of food consumption on weekdays (school day) as opposed to non-school days in both sexes.

The development of nutritional interventions directed at groups of individuals with similar PAs seems to be a promising strategy for allowing greater specificity in actions. For example, children in “Mixed” PA could be encouraged to decrease consumption of high-fat and industrialized foods and substitute these with healthier foods such as fruits and vegetables. On the other hand, the focus of intervention for the schoolchildren in the “Traditional” pattern would be to maintain the healthy aspects of traditional Brazilian foods. In addition, in order to reduce the high prevalence of overweight observed in the present study, related health interventions must also include programs to increase physical activity levels.

## Supporting information

S1 Table Model-fit indexes for latent profile models according to survey year. (DOC)

S2 Table Annual change over the 2013–2015 period for average frequency of consumption (AFC) and ratio to average frequency of consumption by food items according to latent profile DPs of children and adolescents in Florianopolis, Brazil. (DOC)

S1 Dataset Dataset. (XLSX)
