# Supplementary material for: Empirically derived dietary patterns through latent profile analysis among Brazilian children and adolescents from Southern Brazil, 2013-2015
Source: PLoS One. 2019 Jan 8;14(1):e0210425. doi: 10.1371/journal.pone.0210425 (PMC6324812; doi:10.1371/journal.pone.0210425)
Supplement: S1 Table — (DOC) [file pone.0210425.s001.doc]

Table S1. Model-fit indexes for latent profile models according to survey year

| **Number of latent profiles** | **AIC** | **BIC** | **SS-ABIC** | **LMR prob** |
| --- | --- | --- | --- | --- |
|  | **2013** | | | |
| **1** | 85134 | 85312 | 85210 | - |
| **2** | 84158 | 84520 | 84313 | <0.001 |
| **3** | 83522 | 84068 | 83756 | <0.001 |
| **4** | 83127 | 83856 | 83440 | 0.091 |
|  | **2014** | | | |
| **1** | 66627 | 66796 | 66695 | - |
| **2** | 65856 | 66202 | 65996 | <0.001 |
| **3** | 65503 | 66025 | 65714 | 0.011 |
| **4** | - | - | - | 1.000 |
|  | **2015** | | | |
| **1** | 82958 | 83136 | 83034 | - |
| **2** | 81977 | 82337 | 82131 | <0.001 |
| **3** | 81443 | 81987 | 81676 | <0.001 |
| **4** | 83156 | 83884 | 83467 | 1.000 |
|  | **Pooled** | | | |
| **1** | 234725 | 234936 | 234834 | - |
| **2** | 231946 | 232374 | 232167 | <0.001 |
| **3** | 230388 | 231034 | 230722 | <0.001 |
| **4** | - | - | - | 1.000 |

AIC, Akaike information criterion; BIC, Bayesian information criterion; SS-ABIC, Sample size-adjusted Bayesian Information Criterion; LMR prob, Lo-Mendell-Rubin test probability;

-, not available
