# Supplementary material for: Empirically derived dietary patterns through latent profile analysis among Brazilian children and adolescents from Southern Brazil, 2013-2015
Source: PLoS One. 2019 Jan 8;14(1):e0210425. doi: 10.1371/journal.pone.0210425 (PMC6324812; doi:10.1371/journal.pone.0210425)
Supplement: S2 Table — (DOC) [file pone.0210425.s002.doc]

Table S2. Annual change over the 2013-2015 period for average frequency of consumption (AFC) and ratio to average frequency of consumption by food items according to latent profile DPs of children and adolescents in Florianopolis, Brazil.

|  |  | | **Latent dietary patterns [N (% of children)]** | | |
| --- | --- | --- | --- | --- | --- |
|  | Survey year (N) | **Traditional** | | **Monotonous** | **Mixed** |
|  | 2013 (1,942) | [982 (50.6)] | | [560 (28.8)] | [400 (20.6)] |
|  | 2014 (1,520) | [328 (21.6)] | | [833 (54.8)] | [359 (23.6)] |
|  | 2015 (1,902) | [795 (41.8)] | | [656 (34.5)] | [451 (23.7)] |
| Food groups/  or food items/  survey year | **AFC^a^ (95% CI)** | **RAFC^b^ (95% CI)** | | | |
| **Beans (cooked)** | | | | | |
| 2013 | 0.79 (0.76-0.83) | 1.35 (1.29-1.42)* | | 0.34 (0.29-0.39)* | 1.08 (0.98-1.17) |
| 2014 | 0.78 (0.74-0.82) | 1.69 (1.57-1.81)* | | 0.66 (0.61-0.72)* | 1.15 (1.04-1.26)* |
| 2015 | 0.81 (0.78-0.85) | 1.44 (1.36-1.51)* | | 0.39 (0.34-0.44)* | 1.12 (1.03-1.21)* |
| **Cereals** | | | | | |
| Rice | | | | | |
| 2013 | 1.04 (1.00-1.08) | 1.32 (1.27-1.36)* | | 0.43 (0.38-0.49)* | 1.0 (0.92-1.08) |
| 2014 | 1.01 (0.97-1.06) | 1.56 (1.48-1.64)* | | 0.78 (0.74-0.83)* | 0.99 (0.91-1.07) |
| 2015 | 1.01 (0.98-1.05) | 1.40 (1.35-1.45)* | | 0.49 (0.45-0.53)* | 1.03 (0.96-1.11) |
| Manioc flour | | | | | |
| 2013 | 0.22 (0.20-0.25) | 1.18 (1.03-1.33)* | | 0.09 (0.04-0.14)* | 1.95 (1.67-2.24)* |
| 2014 | 0.25 (0.22-0.27) | 1.27 (1.01-1.52)* | | 0.56 (0.46-0.66)* | 1.77 (1.51-2.04)* |
| 2015 | 0.23 (0.21-0.26) | 1.26 (1.09-1.42)* | | 0.16 (0.10-0.23)* | 1.77 (1.51-2.03)* |
| Maize/potatoes | | | | | |
| 2013 | 0.13 (0.11-0.15) | 0.85 (0.68-1.01) | | 0.31 (0.19-0.43)* | 2.38 (1.96-2.81)* |
| 2014 | 0.14 (0.12-0.16) | 0.98 (0.67-1.28) | | 0.59 (0.45-0.74)* | 1.96 (1.56-2.37)* |
| 2015 | 0.12 (0.10-0.13) | 0.73 (0.55-0.90)* | | 0.47 (0.31-0.62)* | 2.26 (1.83-2.69)* |
| Pasta | | | | | |
| 2013 | 0.38 (0.35-0.41) | 0.66 (0.58-0.74)* | | 1.18 (1.04-1.33)* | 1.58 (1.38-1.77)* |
| 2014 | 0.32 (0.29-0.35) | 0.41 (0.28-0.54)* | | 1.00 (0.88-1.12) | 1.54 (1.32-1.76)* |
| 2015 | 0.30 (0.27-0.32) | 0.36 (0.28-0.44)* | | 1.34 (1.18-1.51)* | 1.62 (1.40-1.85)* |
| Instant pasta | | | | | |
| 2013 | 0.15 (0.14-0.17) | 0.60 (0.47-0.73)* | | 1.40 (1.14-1.66)* | 1.60 (1.26-1.94)* |
| 2014 | 0.18 (0.16-0.20) | 0.35 (0.19-0.51)* | | 1.00 (0.84-1.17) | 1.59 (1.27-1.90)* |
| 2015 | 0.18 (0.16-0.19) | 0.49 (0.37-0.61)* | | 1.25 (1.05-1.45)* | 1.54 (1.26-1.82)* |
| Bread/biscuits | | | | | |
| 2013 | 1.09 (1.04-1.13) | 1.2 (1.15-1.26)* | | 0.72 (0.66-0.79)* | 0.87 (0.79-0.95)* |
| 2014 | 1.03 (0.98-1.07) | 1.40 (1.30-1.49)* | | 0.90 (0.84-0.95)* | 0.88 (0.79-0.96)* |
| 2015 | 1.06 (1.02-1.10) | 1.25 (1.18-1.31)* | | 0.76 (0.71-0.82)* | 0.91 (0.83-0.99)* |
| Breakfast cereal | | | | | |
| 2013 | 0.16 (0.14-0.18) | 0.63 (0.50-0.75)* | | 0.75 (0.55-0.95)* | 2.25 (1.87-2.63)* |
| 2014 | 0.17 (0.14-0.19) | 0.64 (0.40-0.88)* | | 0.69 (0.55-0.84)* | 2.04 (1.68-2.41)* |
| 2015 | 0.18 (0.17-0.20) | 0.46 (0.34-0.57) | | 0.79 (0.61-0.96)* | 2.27 (1.96-2.59)* |
| Porridge | | | | | |
| 2013 | 0.07 (0.06-0.08) | 0.86 (0.63-1.08) | | 0.71 (0.43-0.99)* | 1.86 (1.33-2.39)* |
| 2014 | 0.07 (0.06-0.09) | 0.96 (0.55-1.37) | | 0.54 (0.36-0.72)* | 2.1 (1.43-2.76)* |
| 2015 | 0.09 (0.08-0.11) | 1.01 (0.77-1.25) | | 0.64 (0.43-0.84)* | 1.51 (1.11-1.91)* |
| **Meats/eggs/fish and sea foods** | | | | | |
| Beef/poultry | | | | | |
| 2013 | 0.83 (0.80-0.87) | 1.20 (1.14-1.27)* | | 0.63 (0.56-0.69)* | 1.00 (0.91-1.09) |
| 2014 | 0.80 (0.76-0.84) | 1.33 (1.22-1.44)* | | 0.86 (0.80-0.92)* | 1.03 (0.92-1.13) |
| 2015 | 0.77 (0.73-0.80) | 1.20 (1.13-1.27)* | | 0.67 (0.60-0.73)* | 1.13 (1.04-1.23)* |
| Eggs | | | | | |
| 2013 | 0.14 (0.13-0.16) | 1.07 (0.88-1.27) | | 0.43 (0.29-0.57)* | 1.71 (1.38-2.05)* |
| 2014 | 0.17 (0.15-0.19) | 1.14 (0.86-1.42) | | 0.52 (0.39-0.65)* | 1.99 (1.64-2.33)* |
| 2015 | 0.19 (0.17-0.21) | 0.96 (0.79-1.13) | | 0.58 (0.44-0.72)* | 1.68 (1.38-1.97)* |
| Fish/seafood | | | | | |
| 2013 | 0.12 (0.10-0.13) | 0.75 (0.59-0.91)* | | 0.67 (0.45-0.88)* | 1.92 (1.51-2.33)** |
| 2014 | 0.11 (0.09-0.12) | 0.94 (0.57-1.32) | | 0.53 (0.37-0.69)* | 2.14 (1.67-2.62)* |
| 2015 | 0.12 (0.11-0.14) | 0.89 (0.70-1.08) | | 0.62 (0.44-0.81)* | 1.74 (1.39-2.09)* |
| **Fruits/vegetables** | | | | | |
| Fruits | | | | | |
| 2013 | 0.19 (0.17-0.21) | 0.53 (0.42-0.63)* | | 1.21 (0.99-1.43) | 1.84 (1.53-2.15)* |
| 2014 | 0.19 (0.16-0.21) | 0.76 (0.52-0.99)* | | 0.78 (0.62-0.93)* | 1.74 (1.40-2.08)* |
| 2015 | 0.19 (0.17-0.22) | 0.41 (0.31-0.52)* | | 1.16 (0.97-1.35) | 1.80 (1.51-2.10)* |
| Vegetables | | | | | |
| 2013 | 0.21 (0.18-0.23) | 1.05 (0.90-1.20) | | 0.14 (0.08-0.21)* | 2.00 (1.68-2.32)* |
| 2014 | 0.24 (0.21-0.27) | 2.37 (2.03-2.72)* | | 0.25 (0.19-0.32)* | 1.47 (1.23-1.72)* |
| 2015 | 0.23(0.21-0.25) | 1.46 (1.28-1.64)* | | 0.11 (0.06-0.16)* | 1.48 (1.24-1.71)* |
| Green leaves | | | | | |
| 2013 | 0.22 (0.19-0.24) | 1.18 (1.03-1.33)* | | 0.05 (0.02-0.07)* | 1.91 (1.62-2.20)* |
| 2014 | 0.24 (0.21-0.27) | 2.53 (2.19-2.87)* | | 0.20 (0.14-0.26)* | 1.45 (1.21-1.70)* |
| 2015 | 0.22 (0.20-0.24) | 1.50 (1.31-1.69)* | | 0.10 (0.05-0.15)* | 1.43 (1.19-1.68)* |
| Vegetable soup | | | | | |
| 2013 | 0.19 (0.17-0.21) | 0.53 (0.42-0.63)* | | 1.21 (0.99-1.43) | 1.84 (1.53-2.15)* |
| 2014 | 0.19 (0.16-0.21) | 0.76 (0.52-0.99)* | | 0.78 (0.62-0.93)* | 1.74 (1.40-2.08)* |
| 2015 | 0.19 (0.17-0.22) | 0.41 (0.31-0.52)* | | 1.16 (0.97-1.35) | 1.80 (1.51-2.10)* |
| **Milk/milk products** | | | | | |
| Milk and coffee | | | | | |
| 2013 | 0.43 (0.40-0.46) | 1.33 (1.21-1.44)* | | 0.40 (0.31-0.48)* | 1.00 (0.85-1.15) |
| 2014 | 0.46 (0.43-0.50) | 1.50 (1.29-1.70)* | | 0.82 (0.73-0.91)* | 0.97 (0.81-1.12) |
| 2015 | 0.44 (0.41-0.47) | 1.35 (1.22-1.47)* | | 0.69 (0.60-0.79)* | 0.84 (0.71-0.96)* |
| Milk | | | | | |
| 2013 | 0.27 (0.24-0.29) | 1.19 (1.04-1.33)* | | 0.37 (0.27-0.47)* | 1.41 (1.18-1.64)* |
| 2014 | 0.20 (0.18-0.23) | 1.45 (1.14-1.76)* | | 0.49 (0.38-0.60)* | 1.78 (1.46-2.09)* |
| 2015 | 0.23 (0.20-0.25) | 0.98 (0.82-1.14) | | 0.49 (0.38-0.61)* | 1.77 (1.49-2.06)* |
| Yoghurt | | | | | |
| 2013 | 0.42 (0.39-0.45) | 0.81 (0.73-0.89)* | | 0.81 (0.69-0.93)* | 1.81 (1.62-2.00)* |
| 2014 | 0.38 (0.35-0.41) | 1.06 (0.88-1.24) | | 0.61 (0.52-0.69)* | 1.86 (1.64-2.09)* |
| 2015 | 0.36 (0.34-0.39) | 0.83 (0.73-0.93)* | | 0.62 (0.51-0.72)* | 1.86 (1.64-2.07)* |
| Cheese | | | | | |
| 2013 | 0.12 (0.10-0.14) | 1.00 (0.79-1.21) | | 0.17 (0.07-0.26)* | 2.17 (1.68-2.66)* |
| 2014 | 0.12 (0.10-0.14) | 1.45 (1.05-1.84)* | | 0.60 (0.44-0.76)* | 1.53 (1.14-1.91)* |
| 2015 | 0.13 (0.11-0.15) | 1.19 (0.95-1.42) | | 0.29 (0.17-0.40)* | 1.71 (1.36-2.06)* |
| **Salted snacks/fast-foods** | | | | | |
| Cheese bread | | | | | |
| 2013 | 0.14 (0.12-0.16) | 0.36 (0.25-0.47)* | | 0.64 (0.46-0.82)* | 2.93 (2.51-3.35)* |
| 2014 | 0.13 (0.11-0.15) | 0.82 (0.55-1.08) | | 0.38 (0.26-0.50)* | 2.61 (2.12-3.10)* |
| 2015 | 0.12 (0.11-0.14) | 0.43 (0.30-0.57)* | | 0.52 (0.37-0.68)* | 2.69 (2.24-3.14)* |
| French fries | | | | | |
| 2013 | 0.23 (0.21-0.26) | 0.26 (0.19-0.33)* | | 0.83 (0.66-0.99)* | 3.13 (2.77-3.49)* |
| 2014 | 0.23 (0.20-0.26) | 0.13 (0.05-0.21)* | | 0.54 (0.44-0.64)* | 2.86 (2.51-3.21)* |
| 2015 | 0.22 (0.20-0.25) | 0.33 (0.25-0.42)* | | 0.52 (0.41-0.64)* | 2.87 (2.54-3.20)* |
| Chips | | | | | |
| 2013 | 0.11 (0.09-0.12) | 0.27 (0.17-0.38)* | | 0.82 (0.59-1.05) | 2.73 (2.25-3.21)* |
| 2014 | 0.11 (0.09-0.12) | 0.11 (0.00-0.23)* | | 0.57 (0.41-0.74) | 2.8 (2.31-3.28)* |
| 2015 | 0.10 (0.08-0.11) | 0.23 (0.13-0.34)* | | 0.57 (0.39-0.76) | 2.98 (2.47-3.48)* |
| Pizza/hamburger/hot-dog | | | | | |
| 2013 | 0.25 (0.23-0.27) | 0.28 (0.21-0.35)* | | 1.64 (1.44-1.84) | 1.84 (1.58-2.10)* |
| 2014 | 0.24 (0.22-0.27) | 0.04 (0.00-0.08)* | | 1.11 (0.96-1.27) | 1.62 (1.35-1.88)* |
| 2015 | 0.23 (0.21-0.25) | 0.17 (0.11-0.23)* | | 1.57 (1.36-1.77) | 1.64 (1.39-1.89)* |
| Sausages | | | | | |
| 2013 | 0.25 (0.23-0.27) | 0.92 (0.79-1.05) | | 0.56 (0.44-0.68)* | 1.84 (1.57-2.11)* |
| 2014 | 0.27 (0.24-0.30) | 0.78 (0.59-0.98) | | 0.78 (0.65-0.90)* | 1.72 (1.43-2.01)* |
| 2015 | 0.25 (0.23-0.27) | 0.86 (0.72-1.01) | | 0.77 (0.63-0.92)* | 1.57 (1.32-1.81)* |
| Nuggets | | | | | |
| 2013 | 0.06 (0.05-0.07) | 0.00 (0.00-0.07)* | | 1.00 (0.64-1.36) | 3.17 (2.42-3.92)* |
| 2014 | 0.05 (0.03-0.06) | 0.07 (0.00-0.19)* | | 0.57 (0.32-0.81) | 2.86 (1.96-3.77)* |
| 2015 | 0.06 (0.05-0.07) | 0.63 (0.40-0.87)* | | 0.55 (0.31-0.78) | 2.31 (1.74-2.88)* |
| **Sweets** | | | | | |
| Cake | | | | | |
| 2013 | 0.30 (0.28-0.33) | 0.63 (0.54-0.73)* | | 0.93 (0.76-1.10) | 2.00 (1.76-2.24)* |
| 2014 | 0.30 (0.27-0.32) | 0.82 (0.63-1.00) | | 0.72 (0.62-0.83) | 1.81 (1.58-2.04)* |
| 2015 | 0.27 (0.24-0.29) | 0.67 (0.55-0.78)* | | 0.89 (0.74-1.03) | 1.75 (1.51-1.99)* |
| Candies/chocolate/lollypops/ice cream | | | | | |
| 2013 | 0.23 (0.21-0.26) | 0.61 (0.51-0.71)* | | 0.96 (0.79-1.13) | 2.13 (1.85-2.41)* |
| 2014 | 0.24 (0.22-0.27) | 0.60 (0.41-0.79)* | | 0.82 (0.69-0.95) | 1.78 (1.52-2.04)* |
| 2015 | 0.23 (0.21-0.25) | 0.43 (0.33-0.53)* | | 0.95 (0.79-1.11) | 2.07 (1.79-2.35)* |
| Cream cookies | | | | | |
| 2013 | 0.48 (0.45-0.51) | 0.79 (0.71-0.87)* | | 0.96 (0.84-1.08) | 1.54 (1.37-1.71)* |
| 2014 | 0.47 (0.43-0.50) | 0.69 (0.56-0.82)* | | 0.73 (0.65-0.82)* | 1.90 (1.69-2.11)* |
| 2015 | 0.43 (0.40-0.46) | 0.67 (0.58-0.76)* | | 0.78 (0.68-0.88)* | 1.91 (1.72-2.09)* |
| **Sugar-sweetened beverages** | | | | | |
| Chocolate milk | | | | | |
| 2013 | 0.48 (0.44-0.51) | 0.83 (0.75-0.92)* | | 1.04 (0.92-1.16) | 1.29 (1.13-1.45)* |
| 2014 | 0.45 (0.41-0.49) | 0.69 (0.54-0.85)* | | 0.90 (0.79-1.00) | 1.52 (1.32-1.71)* |
| 2015 | 0.48 (0.45-0.52) | 0.82 (0.73-0.91)* | | 0.96 (0.84-1.07) | 1.38 (1.23-1.53)* |
| Fruit juices | | | | | |
| 2013 | 0.54 (0.51-0.58) | 0.98 (0.89-1.08) | | 0.52 (0.43-0.61) | 1.74 (1.55-1.93)* |
| 2014 | 0.56 (0.52-0.60) | 1.38 (1.19-1.57)* | | 0.68 (0.60-0.77) | 1.38 (1.21-1.56)* |
| 2015 | 0.54 (0.51-0.58) | 0.97 (0.86-1.07) | | 0.68 (0.58-0.78) | 1.53 (1.36-1.69)* |
| Sodas | | | | | |
| 2013 | 0.51 (0.48-0.55) | 0.51 (0.44-0.58)* | | 1.24 (1.09-1.38) | 1.90 (1.71-2.09)* |
| 2014 | 0.51 (0.47-0.55) | 0.31 (0.23-0.40)* | | 0.88 (0.78-0.98) | 1.91 (1.72-2.09)* |
| 2015 | 0.50 (0.47-0.54) | 0.39 (0.32-0.45)* | | 1.03 (0.91-1.15) | 2.04 (1.85-2.23)* |

AFC: Average Frequency of Consumption; RAFC: Ratio to Average Frequency of Consumption

^a^ AFC: Sample average frequency of consumption

^b^ RAFC: The ratio of the mean food intake among the children belonging to each latent pattern and the AFC

* When 95% confidence interval does not include the value of one, it is statistically significant.
